# Registration for Children's Trained Nurses: A Matron's View

**Published:** 1919-03-01

**Authors:** M. P. Ferrier


					Registration for Children's Trained Nurses: A Matron's View.
To the Editor of The Hospital.
Sir,?Will you kindly correct the error in the heading
of my letter to The Hospital of February 15? I am not
a children's nurse nor have I been trained in a children's
hospital. For some years, besides my ordinary work,
I have had to deal with large numbers of/ children, and
my nursing staff consisted of some trained in general
work, and others in children's only, therefore I fee]
qualified to compare and give an opinion on the two
systems; but mine: is only a suggestion as a t possible
way out of a difficulty.?Yours truly,
M. P. Merrier.

				

